# Supplementary material for: Crawling and Gliding: A Computational Model for Shape-Driven Cell Migration
Source: PLoS Comput Biol. 2015 Oct 21;11(10):e1004280. doi: 10.1371/journal.pcbi.1004280 (PMC4619082; doi:10.1371/journal.pcbi.1004280)
Supplement: S1 Code — (ZIP) [file pcbi.1004280.s012.zip › release/tst/doc/html/namespaceColourMode.html]

Tissue Simulation Toolkit: ColourMode Namespace Reference


Tissue Simulation Toolkit
 0.1.4.1


- Main Page
- Namespaces
- Classes
- Files

- Namespace List
- Namespace Members

Enumerations

ColourMode Namespace Reference

|  |  |
| --- | --- |
| Enumerations | |
| enum | { State, CellType, Sigma, Auxilliary } |
|  | |

## Enumeration Type Documentation

anonymous enum

| Enumerator | |
| --- | --- |
| *State* |  |
| *CellType* |  |
| *Sigma* |  |
| *Auxilliary* |  |


---

Generated on Thu Aug 14 2014 22:04:01 for Tissue Simulation Toolkit by  

 1.8.6
